# Family structure and sociodemographic factors associated with molar incisor hypomineralization in children

**DOI:** 10.1038/s41598-026-39879-5

**Published:** 2026-02-11

**Authors:** Sílvia Lluís Poy, Ana Veloso Durán, José Fernández Sáez, Alba Romero Martinez, Carla Juárez Fusté, Francisco Guinot Jimeno

**Affiliations:** 1 Department of Paediatric Dentistry, Universitat Internacional de Catalunya (UIC), Barcelona, Spain; 2 Servei d’Atenció Primària Terres de l’Ebre, Institut Català de la Salut (ICS), Tortosa, Spain; 3 Fundació Institut Universitari per a la Recerca a l’Atenció Primària de Salut Jordi Gol I Gurina (IDIAPJGol), Barcelona, Spain; 4 School of Nursing, Rovira i Virgili University, Terres de l’Ebre Campus, Tortosa, Spain; 5 Service of Pediatric Dentistry, Hospital HM Nens, HM Hospitales, Barcelona, Spain; 6 HM Hospitals Health Research Institute, Madrid, Spain; 7 Department of Paediatric Dentistry, Faculty of Dentistry, Universitat Internacional de Catalunya (UIC), St. Cugat del Vallès (Barcelona), Spain Josep Trueta s/n, 08190

**Keywords:** Molar incisor hypomineralization, Family structure, Sociodemographic factors, Diseases, Health care, Medical research

## Abstract

Molar Incisive Hypomineralization (MIH) is a condition in the dental enamel of multifactorial etiology and, in part unknown. This study aimed to determine if there are coincident family or sociodemographic patterns in the group of pediatric patients diagnosed with MIH. A cross-sectional descriptive observational study. An oral examination was performed to diagnose the presence or absence of MIH in children aged 6 to 14 years who attended the Dentistry Service of the Primary Care Center in Roquetes, Catalunya, Spain, plus a questionnaire created ad hoc with sociodemographic and family structure variables that the parents filled out during the visit. A total sample of 305 patients was recruited; 153 had MIH and 152 did not. The sample was grouped, according to the country of maternal origin: (1) children with a mother of Spanish nationality (44.92%), and (2) children with a mother of foreign nationality (55.08%). In group 1, a significant association was found between having MIH and the variables “Mother with higher education” (p-value = 0.002); “Child does not live with both parents” (p-value = 0.012); “Place of birth of the child other than the place of current residence” (p-value = 0.002). In group 2, the variables correlated with MIH were: “Mother’s locality different from the father’s locality” (p-value = 0.002); “Mother’s locality different from the child’s birth” (p-value = 0.001). Unfavorable sociodemographic factors, the phenomenon of migration, and the family structure of not living with both parents were associated with the presence of MIH in the children in the present study.

**Supplementary Information:**

The online version contains supplementary material available at 10.1038/s41598-026-39879-5.

## Introduction

Molar Incisive Hypomineralization (MIH) is a clinical picture with a worldwide prevalence ranging from 2.4 to 40.2%^1,2^. It is estimated that the average prevalence is 13.5%, with moderate to severe involvement in 36% of patients with MIH and, in 36.6% of cases, with incisor involvement. In Spain, there are heterogeneous analyses, with their estimated prevalence, depending on the different studies^2–4^, ranging between 14%^4^ and 21%, varying according to the regions^3^. This wide variability may reflect methodological differences across studies rather than true epidemiological disparities. Its etiology, according to studies carried out so far^1,5–9^, is multifactorial and, in turn, unknown. It has been observed that there is a correlation between certain diseases in early childhood, or conditions during pregnancy, and the occurrence of MIH, suggesting that there are physiological stressors that can alter the function of ameloblasts^1,7^. The hypoxia and pyrexia that they may suffer in case of fever or other adverse systemic circumstance in certain stages, can generate this clinical picture. Genetic predisposition and the role of epigenetic influences are increasingly present in terms of their etiology^7^.

Taking into account the World Health Organization’s (WHO) definition of health, as “a complete state of physical, mental and social well-being”, it is considered that there is an interaction between social support and health: a greater availability of interpersonal contacts means greater social support; and, in turn, people who perceive less social support are more predisposed to experience emotional and physical disorders when they face high levels of stress. compared to people who have that support^10^. In this sense, the housing factor (residential stability) has been proven to directly and indirectly affect health in various ways and represents one of the key social determinants ofhealth^11^.

The family is the element of the structure of society responsible for the biological and social reproduction of the human being; it is the element that synthesizes the production of health at the microsocial scale. In their early chilhood, supportive family environments play a relevant role in child development and health In the family nucleus, the needs that are at the basis of the conservation, promotion and recovery of health are satisfied. Therefore, the family plays important roles in the biological, psychological, and social development of the individual^12^.

The roles of the mother and father figure in terms of the couple relationship and domestic organization, as well as social and family aspects, are fundamental in the health and adequate development of children. Assuming that MIH triggers act in the period between pregnancy and early childhood^1,6,7,9^, it is critical to consider the influence that maternal health plays on child well-being^13,14^.

There is evidence that a situation of parental conflict^15^, a lack of maternal social support^13^ or residential instability^11^ are factors that can have an impact on maternal health, generating a difficulty in the psychological development of the child and, in turn, negatively affecting his health^15^.

There is little evidence in the current literature to support a causal relationship between sociodemographic and family factors and MIH. Besides, potential contexts, such as access to healthcare, nutrition, environmental exposures, or genetic susceptibility are factors that may interact with both social and family factors.

The aim of this study was to assess whether certain family patterns and maternal sociodemographic aspects are related to the appearance of the clinical picture of MIH in the patients studied.

## Material and method

### Study design and population

An observational, cross-sectional with a comparison group study was conducted to evaluate the associations between sociodemographic and family factors and the presence of MIH in children seen between May and October 2024 in the dentistry clinic of the Primary Care Center (CAP) of Roquetes, in Terres de l’Ebre, Tarragona, Spain. This CAP belongs to the Catalan Institute of Health (ICS) and serves part of the population of the Tortosa Oest Basic Health Area (ABS), within the Terres de l’Ebre Health Region.

The Ethics Committee of the Jordi Gol i Gorina Foundation approved the study with the CEIm code: 24/051-P in May 2024. Inorder to obtain a representative sample, assuming an alpha risk of 5% and a beta risk of 20%, the minimum participation of 304 patients (152 with MIH and 152 as a control group, without MIH) was calculated as necessary.

The inclusion criteria for the sample were those patients aged between 6 and 14 years who attended the dentistry clinic of CAP Roquetes and agreed to participate in the study. Patients who had amelogenesis imperfecta or fluorosis, or who did not have erupted permanent first molars were excluded from the study.

The study was conducted in accordance with the Declaration of Helsinki (last updated in October 2013) and the International Conference on Harmonisation (ICH) Good Clinical Practice (GCP) guidelines.

### Procedure

Data collection and subsequent processing was performed by a single examiner (SLP), a dentist specialized in paediatric dentistry and whith more than 20 years of experience as a dentist in public heath. Also that dentist was trained in the detection of MIH according to the Standardized Guidelines for the Diagnosis and Recording of MIH introduced by Ghanim et al. (2015) and referenced by the EAPD^16^, considering as MIH lesions the demarcated opacities, white, yellow or brown, which may or may not present caries lesions or atypical fillings, with or without enamel rupture, present in 1 or more first permanent molars, with or without involvement of the permanent incisors and, ruling out other local or systemic enamel lesions, not MIH compatible. Intra-examiner calibration was performed through 50 photographs of cases with MIH, cases of caries lesions in permanent molars and cases of healthy molars, obtaining an intra-examiner Kappa index = 1.

Patients aged 6 to 14 years who attended the dentistry clinic at the CAP Roquetes health centre were proposed by the same dentist to participate in the study. The characteristics of the project were explained to the family, giving them an explanatory sheet on the motivation of the child and the steps that would be followed; If they agreed to participate, they were given an informed consent form and a questionnaire created for this study, mostly qualitative and multiple-test, which consisted of 11 questions; in the first 2, the variables of family structure were collected, in the next 7 questions, data related to the sociodemographic variables were collected, and in the last 2, data were collected for the socioeconomic variable of the family. The questionnaire was created for this study in order to collect sociodemographic and family structure data (Annex 1). It was delivered and filled in by the parents on the same day of the appointment, when the same dentist made the treatment or visit, making the diagnosis of MIH, and registering that both in the questionnarie and the medical record.

Taking into account that in this study the link between the patient and the mother was considered, with maternal health and well-being being a conditioning factor in the child during pregnancy and early childhood, the sociodemographic and family variables that directly affected the mother were taken into account; that is why, based on the nationality of the child’s mother, a dichotomous variable was created to classify the children’s mothers as Spanish or foreign. A Spanish mother was considered to be one who spent her childhood in Spain, and within Spanish sociocultural background.

The clinical data and those obtained in the questionnaire were recorded by coding, in accordance with the Data Protection Law, in an Excel sheet designed for the study. Once the study questionnaire was completed, the patient’s medical history was accessed through the eCAP computer program, used by the health professionals of the ICS, which records their medical history, clinical course, notes of the actions carried out on the day of the visit, and, in the case of the Dentistry specialty, there is a section within the medical record. the odontogram, where the diagnoses and treatments of the dental activity are recorded, as well as the presence or not of MIH.

### Statistical analysis

A descriptive analysis of the total sample was carried out, stratified by nationality of the child’s mother and whether the child had MIH, using frequency and percentage of the variables. To detect statistically significant differences between the variables depending on whether the child had MIH or not, the Z test of differences in proportions was performed. To quantify this relationship, odds ratios (ORs) and their 95% confidence intervals (95% CIs) were calculated using logistic regression models.

Statistical analyses were performed with IBM’s SPSS.20 statistical package and *p* < 0.05 was taken as a statistical significance level.

## Results

A study sample of 305 children was recruited: 153 diagnosed with MIH and 152 without MIH as a control group. 136 males (44.6%) and 169 females (55.4%), with a mean age of 10.6 years and a standard deviation (SD) of 2.33.

Of the total sample, 44.92% were children of mothers with Spanish nationality and 55.08% of mothers with foreign nationality, with the majority of mothers of Moroccan nationality (41.64%) being in this group. As shown in Table 1, the sample was grouped according to the different variables that were the object of this study: sociodemographic and family structure variables.

**Table 1:** Description of the sample of children in the study according to sociodemographic and family structure variables.

	*n*	%
Total	305	
MIH	153	50,16
Child’s age (mean, ^ads^)	**10**,**6**	**2**,**33**
Mother nationality		
Spain	137	**44**,**92**
Morocco	127	**41**,**64**
Pakistan	8	2,62
Romania	8	2,62
Ecuador	4	1,31
Honduras	4	1,31
Moldova	3	0,98
Brazil	2	0,66
Colombia	2	0,66
Gambia	2	0,66
Mali	2	0,66
Ukraine	2	0,66
Argentina	1	0,33
Cuba	1	0,33
Peru	1	0,33
China	1	0,33
Nationality of maternal origin		
Foreigner	168	55,08
Spaniard	137	44,92
Family structure		
In the 1st year of life he lived with both his parents	280	91,80
She currently lives with both parents	249	81,64
He currently lives only with his mother	34	11,15
She currently lives only with her father	7	2,30
Joint custody	10	3,28
Currently living in a nursing home/shelter	2	0,66
Marital status of the parents		
Parents live together	249	81,64
Separated/divorced/widowed parents	50	16,39
Single-parent family	5	1,64
Adopted child	2	0,66
Socio-demographic factor		
The mother’s place of origin is the same as the father’s	196	64,26
The mother’s place of origin is the same as that of the child’s birth	160	52,46
The child’s place of birth is the same as the child’s current place of residence	190	62,30
He is an only child	29	9,51
The child has 1 sibling	104	34,10
The child has 2 or more siblings	172	56,39
Mother’s age (in years)		
18–25	3	0,98
26–35	66	21,64
36–45	160	52,46
46–65	75	24,59
Primary caregiver studies		
Higher education	110	36,07
Secondary education	92	30,16
Primary education	63	20,66
Not a school graduate	38	12,46
Family income		
< €12,000	74	24,26
12.000–30.000€	196	64,26
> €30,000	36	11,80

^a^Standard deviation.

Initially, the total of 305 patients were grouped into 2 groups according to nationality: 137 patients with a Spanish mother and 168 with a foreign mother, to assess the relationship between the presence or absence of MIH and maternal nationality. 81 of the children of a Spanish mother (59%) and 72 of the children of foreign mother (42’9%) presented MIH. 56 children of Spanish mother (40’9%) and 96 children of foreign mother (57,1%) did not presented MIH. In both cases, statistically significant differences were found in relation to whether or not they had MIH according to maternal nationality (p-value = 0.005) (Table 2).

**Table 2:** Comparison between the relationship between the variable of Spanish or foreign maternal nationality and the presence or absence of MIH, respectively.

	Spanish mothers		Foreign mothers		*p*
	*n*	%	*n*	%	
Total	**137**		**168**		
MIH	81	59,1	72	42,9	**0**,**005**
No MIH	56	40,9	96	57,1	**0**,**005**

Significant values are in bold.

Among the children of Spanish mothers who had MIH, statistically significant differences were found associated with the following variables: “Mother with higher education” (p-value = 0.002); “Child does not live with both parents” (p-value = 0.012); “Separated/divorced/widowed parents” (p-value = 0.020); “Child’s place of birth different from the current place of residence” (p-value = 0.002) (Table 3).

**Table 3:** Description of the sociodemographic and family variables studied, according to the nationality of the mothers and according to whether or not they have MIH.

	Spanish mothers					Foreign mothers				
	MIH		No MIH		*p*	MIH		No MIH		*p*
	*n*	%	*n*	%		*n*	%	*n*	%	
Total	**81**		**56**			**72**		**96**		
Primary caregiver higher education	54	66,7	22	39,3	**0**,**002**	16	22,2	18	18,8	0,579
You do NOT currently live with both parents	34	42,0	12	21,4	**0**,**012**	5	6,9	5	5,2	0,638
Parents do NOT live together	34	42,0	12	21,4	**0**,**012**	5	6,9	5	5,2	0,638
Separated/divorced/widowed parents	31	38,3	11	19,6	**0**,**020**	5	6,9	3	3,1	0,250
Single-parent family	3	3,7	1	1,8	0,512	0	0,0	1	1,0	0,385
Mother locality DIFFERENT father locality	40	49,4	32	57,1	0,371	24	33,3	13	13,5	**0**,**002**
Mother locality DIFFERENT locality birth child	22	27,2	10	17,9	0,206	61	84,7	52	54,2	**< 0.001**
Child’s place of birth DIFFERENT current residence	27	33,3	6	10,7	**0**,**002**	32	44,4	50	52,1	0,327

Significant values are in bold.

The variables in the group of foreign mothers that showed a statistically significant association with MIH were: “Mother’s locality different from the father’s locality” (p-value = 0.002) and “Mother’s locality different from the child’s birthplace (p-value < 0.001). The mother’s age equal to or greater than 46 years behaved as a protective factor in this sample (p-value = 0.039) (Table 4).

**Table 4:** Description of the sociodemographic and family variables studied, according to the nationality of the mothers and according to whether or not they have MIH.

	MIH					No MIH				
	Spanish mothers		Foreign mothers		*p*	Spanish mothers		Foreign mothers		*p*
	*n*	%	*n*	%		*n*	%	*n*	%	
Total	**81**		**72**			**56**		**96**		
Primary caregiver higher education	54	66,7	16	22,2	**< 0.001**	22	39,3	18	18,8	**0**,**006**
You do NOT currently live with both parents	34	42,0	5	6,9	**< 0.001**	12	21,4	5	5,2	**0**,**002**
Parents do NOT live together	34	42,0	5	6,9	**< 0.001**	12	21,4	5	5,2	**0**,**002**
Separated/divorced/widowed parents	31	38,3	5	6,9	**< 0.001**	11	19,6	3	3,1	**0**,**001**
Single-parent family	3	3,7	0	0,0	0,099	1	1,8	1	1,0	0,698
Mother locality DIFFERENT father locality	40	49,4	24	33,3	**0**,**045**	32	57,1	13	13,5	**< 0.001**
Mother locality DIFFERENT locality birth child	22	27,2	61	84,7	**< 0.001**	10	17,9	52	54,2	**< 0.001**
Child’s place of birth DIFFERENT current residence	27	33,3	32	44,4	0,159	6	10,7	50	52,1	**< 0.001**

Significant values are in bold.

When the variables in the group of patients with MIH were compared, depending on whether the mother was of Spanish or foreign nationality, the main statistically significant differences (p-value: < 0.001) were obtained in the following variables: “Main caregiver with higher education”, “Not living with both parents”, “Parents do NOT live together”, “Separated/divorced/widowed parents” and “Different mother locality birth of child” (Table 4).

In relation to the mother’s age, being equal to or greater than 46 years behaved as a protective factor in this sample (p-value = 0.039) (Table 5).

**Table 5:** Description of the relationship between having MIH and the age of the mother.

	Spanish					Foreigner				
	MIH		No MIH			MIH		No MIH		
	*n*	%	*n*	%	*p*	*n*	%	*n*	%	*p*
Total	**82**		**55**			**71**		**96**		
Age of mother										
18–25 a.	0	0,00	0	0,00		1	1,22	2	2,08	0,737
26–35 a.	11	13,41	5	9,09	0,405	25	30,49	25	26,04	0,223
36–45 a.	50	60,98	32	58,18	0,590	35	42,68	43	44,79	0,623
46–65 a.	21	25,61	18	32,73	0,428	10	12,20	26	27,08	**0**,**039**

Significant values are in bold.

**Table 6:** Description of the relationship between the socioeconomic variable and the presence of MIH in the total sample.

	MIH				*p*
	Yes		No		
	*n*	%	*n*	%	
Total	**153**		**152**		
Family income < €12,000	30	19,35	44	29,14	**0**,**046**
Family income €12,000–30,000	106	68,39	90	59,60	0,109
Family income > €30,000	19	12,26	17	11,26	0,786

Significant values are in bold.

Among the results found in the overall sample, low socioeconomic status was related to the presence of MIH, although when the sample was separated according to maternal nationality, the result was no longer statistically significant (Tables 6 and 7).

**Table 7:** Description of the relationship between the socioeconomic variable and the presence of MIH, grouping the sample according to maternal nationality.

	Spanish mother				*p*	Foreign mother				*p*
	MIH					MIH				
	Yes		No			Yes		No		
	*n*	%	*n*	%		*n*	%	*n*	%	
Total	**81**		**56**			**72**		**96**		
Family income < €12,000	10	12,20	10	17,86	0,354	20	27,40	34	35,79	0,248
Family income €12,000–30,000	54	65,85	31	55,36	0,213	52	71,23	59	62,11	0,215
Family income > €30,000	18	21,95	15	26,79	0,513	1	1,37	2	2,11	0,721

Significant values are in bold.

**Fig. 1:**
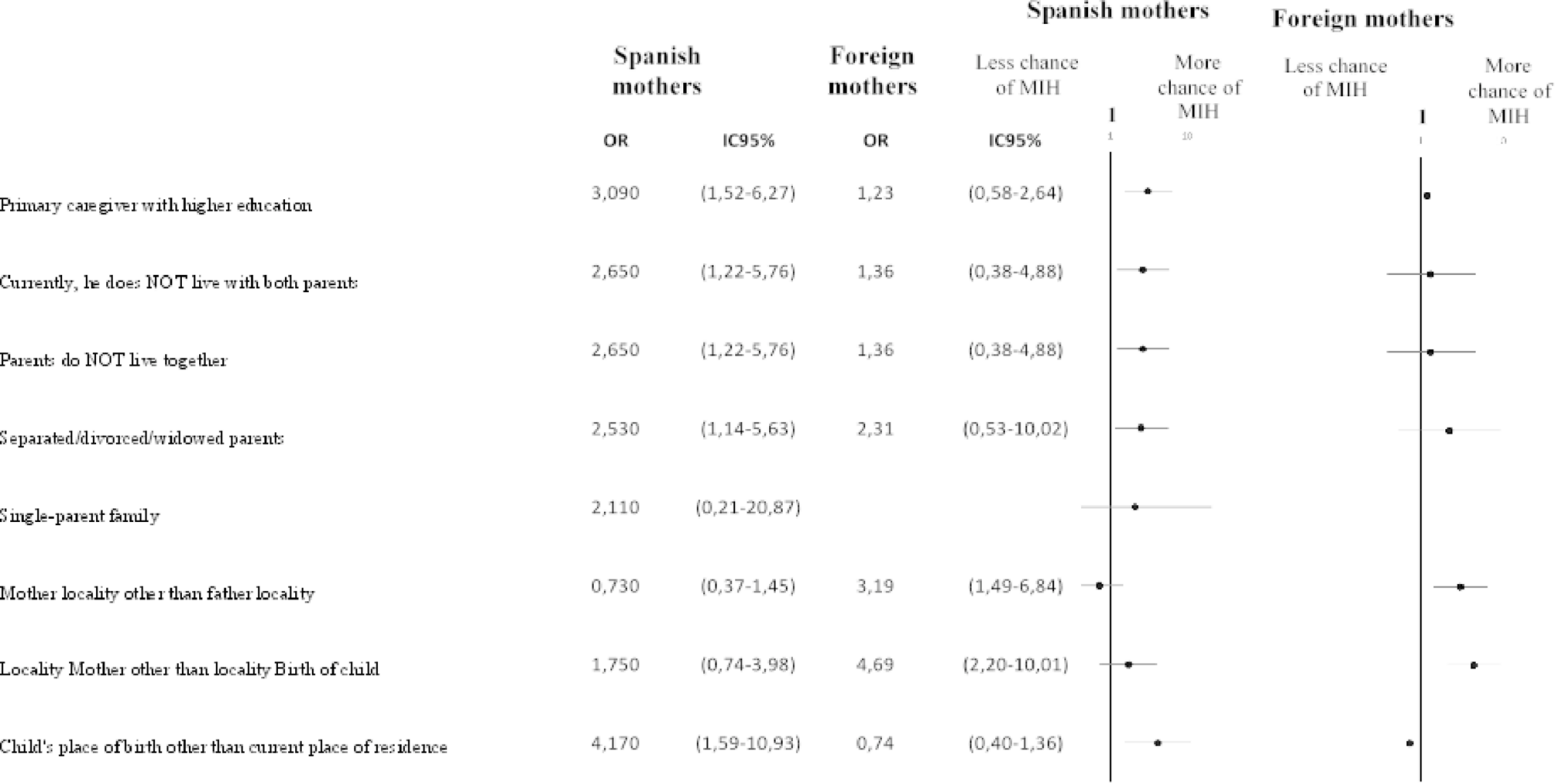
Association between the characteristics of the parents and the presence of MIH in children depending on whether the mother is Spanish or foreign.

Regarding the calculation of the Odds Ratio (OR) of probability of presenting MIH according to the different variables, the following significant results were found (Fig. 1).

The variable “Primary caregiver with higher education” obtained, in the group of mothers of Spanish nationality, a probability OR of 3.09 with a 95% CI of 1.52–6.27, being not significant in the group of foreign mothers. The variable “Currently does not live with the 2 parents” obtained, in the group of mothers of Spanish nationality, an OR of 2.65 with a 95% CI of 1.22–5.76, resulting in non-significant in the group of foreign mothers.

Regarding the variable “Separated/divorced/widowed parents”, in the group of mothers of Spanish nationality obtained an OR of 2.53 with a 95% CI of 1.14–5.63. In the group of foreign mothers, an OR of 2.31 was obtained, and the 95% CI was 0.53–10.02. The variable “Mother’s locality other than father’s locality” obtained an OR of 3.19 in the foreign mother group, with a 95% CI: 1.49–6.84, resulting in non-significant in the group of Spanish mothers. The variable “Mother’s locality other than the child’s place of birth” obtained an OR of 4.69 with a 95% CI: 2.2-10.01 in the group of mother of foreign nationality, being less significant in the group of mother of Spanish nationality (OR: 1.75, 95% CI: 0.74–3.98). Finally, the variable “Child’s place of birth other than current place of residence” obtained, in the group of mothers of Spanish nationality, an OR of 4.17, with a 95% CI of 1.59–10.93, while in the group of mothers of foreign nationality, it did not obtain significant results.

## Discussion

When we analyze the current literature on the etiology of MIH, the factors most commonly related to its occurrence are a history of illness during pregnancy, low birth weight, general illness in childhood, antibiotic use, and high fever during early childhood^1,6,7,9^. Currently, there is little evidence about the impact of sociodemographic factors and family structure on the occurrence of MIH. In the present study, taking into account the description of health from the social perspective and focusing on the family nucleus as the basic structure of the individual, an association has been found between certain maternal sociodemographic factors and specific family structures with the presence of this clinical picture.

We found a statistically significant difference (p-value = 0.005) regarding the presence of MIH when we compared the group of children with a Spanish mother with that of a foreign mother, with the presence of MIH being more prevalent in children of a Spanish mother than in those of a foreign mother. These results are consistent with the estimated prevalence of MIH in Spain (14–21%) and Africa (11.6%), taking into account that there are still no studies with sufficient sample and scientific strength to evaluate the prevalence of MIH in the Moroccan population^17^.

In the case of families with a foreign mother, the sociodemographic factors associated with MIH were: the mother’s locality other than the father’s locality, and the mother’s locality other than the child’s birth. The mother’s age greater than or equal to 46 years was associated with lower odds of MIH. A possible hypotesis to associate with that results could be the fact that this population analyzed has suffered a separation from their close family environment due to the fact of changing their place of residence due to the migratory phenomenon; There is a process called “acculturation”, which explains why immigrants see their state of health deteriorate when they arrive in their country of destination, due to the fact that they are uprooted from their social network, a phenomenon that affects immigrant women in greater proportion. In addition, the Moroccan family model (population that makes up 41.64% of the sample of our study) must be taken into account. There is the traditional Moroccan family model or the current one, which has emerged as a result of adapting to the changes in contemporary society, with greater autonomy for women and an increase in families whose members emigrate^18,19^; in both models, their main characteristic is a strong cohesion, structure and hierarchy between family members. the family being the main social structure on which their education and vital dynamics are based. It is logical then that the fact that the mother is over 46 years of age could be a a factor related to a lower probability of MIH, since it is inferred that when the mother had the baby, she had already been in the new country of residence for some time, having adapted to the environment and having woven a social network and a new family structure^18^. This is consistent with the fact that the immigrant population, with the time of residence, can adapt to the preventive strategies of our country, improving access to treatment for the benefit of their health^20^; taking into account, in addition, that the Moroccan immigrant group is in good health in proportion to the “time of residence” factor, compared to other groups^19^.

Although there is no full consensus in the current literature regarding the influence of education levels on health, our result regarding the variable “mother with higher education” is consistent with part of the existing literature, where it is considered that higher education can be a stressor^21^. Although a higher level of education is considered to be a protective factor in terms of the individual’s longevity, it has also been detected that the level of stress and demand of higher education could have an impact on greater psychological pressure, which can predispose to less personal care in terms of diet and physical exercise. and have an impact on a worse state of psychological health^22^, generating stress that, in the case of the maternal figure, could be a factor that can affect the baby’s health and lead to its deterioration^13^.

On the other hand, the variable “child’s place of birth other than the current place of residence” is associated with the residential instability factor, being consistent with the literature where children’s residential mobility and its negative relationship with their health are associated^23^and, in turn, is consistent with the literature that suggests residential instability as an important maternal stressor^24^.

The variables of non-cohabitation of the child with his two parents and parental separation associated with MIH are consistent with different studies in which family conflicts are related to the presence of MIH^5,25^; in the same way that parental separation is related to the deterioration of child health.

We can understand that there is a relationship between residential stability, cohabitation with both parents, and their separation; although we have analyzed the variables separately, we assume that there may be overlap and collinearity between them.

The Spanish family model is essentially different from the foreign population studied; it has undergone a series of modifications, especially today, characterized by a tendency towards a society in which individuality is more valued, the diversification of the family structure or model with gender policies and with modifications in marriage and child custody laws^26^; For this same reason, it is more likely to find in our country very heterogeneous families in terms of their structure and with maternal roles with a significant labor and economic weight and not only as caregivers of their children in the family home.

In both the group of Spanish mothers and the group of foreign mothers in this study, the factors mentioned above have in common an alteration of the maternal family structure; that is, in families with a foreign mother, there is a separation from her family nucleus and, as for the Spanish mother, there is a breakdown of the family structure (due to the separation of the parents or due to the change of mother-child residence). There are numerous references in the literature as to how stress or a maternal depressive state can affect the health of the baby in the postpartum period; their social support and family being determining factors^10,13,14,25^; In fact, when there is parental conflict between the parents, it can affect the child’s physical, emotional, psychological, and social health, increasing their anxiety and even presenting a greater inflammatory response^14,15^. Both the breakdown of the family structure and the separation from her family or social network are disruptive factors that could cause a stressful situation for a mother, and this maternal stress can be associated with the appearance of the clinical picture of MIH in her children.

Recent studies have associated maternal stress with the occurrence of MIH^11,12,27^, although more longitudinal studies are needed to draw clear conclusions about this. Also, the association between maternal stress and the respiratory pathology of the baby has been recently confirmed^28^, including a greater tendency of the latter to suffer from infectious diseases^29^; these pathologies may present with clinical pictures considered as etiological factors of MIH. It should be mentioned that maternal depression has been associated prior to or during pregnancy with an alteration in the mineralization of the enamel in the primary dentition, causing an increase in the neonatal line^30^; these hypotheses being consistent with the results of this study, although it should be noted that the mechanisms by which maternal stress can affect the baby’s health and the presence of MIH are currently under investigation and more studies are needed to draw firm conclusions. Therefore, taking into account that stress can be an aggravating factor of pathology^10,13,14,30^ and that a family breakdown^14^ or residential instability can enhance stress^11^, it is advisable to evaluate the clinical picture of MIH from a more global perspective in order to find, within its multifactorial etiology, a common factor that can predict the appearance of MIH and, thus, to be able to apply more efficient preventive measures^5^.

When we look at the socioeconomic factor, analyzing the total sample, we see that there is a statistically significant association between presenting MIH and the variable “Annual family income less than 12,000” (p-value = 0.046). These results are consistent with results from previous studies linking MIH to low household income^31,32^. This socioeconomic factor associated with MIH can be explained by maternal stress generated by an unfavorable economic situation; often associated with residential instability, a situation that, as we have mentioned above, can generate a deterioration of maternal health and affect the health of the baby. However, when we stratify according to maternal nationality (Spanish or foreign), this association is no longer statistically significant. Probably, the explanation for these results is related to the size of the sample; when we stratify according to nationality it is reduced and the result loses statistical weight, or also due to the modification of the effect or residual confounding.

This study has strengths and limitations. The limitations are especially in the sample, which belongs to a rural area of Catalonia, with specific sociodemographic characteristics and composed mostly of patients with dental pathologies who were referred to the fillings portfolio service (a public service run by the ICS, through which fillings are performed in permanent dentition of children aged 6 to 14 years in primary care centers of reference).

Another limitation to consider is that the biological, physiological, and genetic etiological variables of MIH were not recorded, so they could represent unassessed confounding factors. Due to the cross-sectional nature of the study, it was not possible to objectively evaluate the maternal stress factor, only to infer it from the data obtained. Moreover, the data collection questionnaire was not previously validated, but rather created specifically for this study.

The strengths of this study were that data collection was performed by a single examiner, thus eliminating inter-examiner bias; In turn, to eliminate the recall bias, subjective questions were eliminated from the questionnaire, providing concrete information from which it can be inferred whether there was any change in the family structure or in their sociodemographic status that could be significant. In order to provide more light on this topic, a multicenter and longitudinal study would be necessary, starting during pregnancy, measuring the variables of emotional well-being and social support with validated tools, recording the timing of migration, and using multilevel or mixed approaches to obtain an adequate record of social determinants, and to assess, a posteriori, whether there is MIH in their children, and mesure the severity.

## Conclusions

- Some specific sociodemographic factors, like the phenomenon of migration and the family structure of not living with both parents were associated with the presence of Incisive Molar Hypomineralization (MIH) in the children in the present study.
- The relationships between the sociodemographic and family variables studied and the presence of MIH showed a different pattern depending on maternal nationality.
- The change in the maternal family structure, within their social context, was associated with the presence of MIH in the patients in this study.

## Supplementary Information

Below is the link to the electronic supplementary material.

Supplementary Material 1

## Data Availability

The datasets generated and/or analysed during the current study were made available to the journal for editorial review. Due to the inclusion of children in the sample, the raw data cannot be made publicly available in order to protect the privacy of minors. All shared data are fully de-identified and may be obtained from the corresponding author upon reasonable request.
