# Supplementary material for: Family structure and sociodemographic factors associated with molar incisor hypomineralization in children
Source: Sci Rep. 2026 Feb 11;16:8301. doi: 10.1038/s41598-026-39879-5 (PMC12966381; doi:10.1038/s41598-026-39879-5)
Supplement: Supplementary file 1 [file 41598_2026_39879_MOESM1_ESM.odt]

Annex 1: Questionnarie

1. Does the child live with both parents?
o Yes
o No

If not, the child lives with:
o Mother
o Father
o Close relatives
o Foster home or residential care center
o Other (specify):

2. Marital status of the parents:
(mark one option)
o Married
o Living together / common-law partnership
o Separated
o Single-parent family
o Adopted child
o Other (specify):

3. Where is Father from (city/town):
4. Where is Mother from (city/town):
5. Child’s place of birth (city/town):
6. Current family residence (city/town):
Since when (year):

7. The child:
o Is an only child
o Has siblings (specify number of siblings):

8. Age of main caregiver:
o 18–25 years
o 26–35 years
o 36–45 years
o 46–65 years
o Over 65 years

9. Sex of main caregiver:
o Male
o Female

10. Educational level of main caregiver:
o Higher education (university or technical training)
o Secondary education
o Primary education
o Other / not graduated from school

11. Annual family income:
o Less than €12,000
o Between €12,000 – €30,000
o More than €30,000
